# Extreme Temperatures and Mortality: Assessing Effect Modification by Personal Characteristics and Specific Cause of Death in a Multi-City Case-Only Analysis

**DOI:** 10.1289/ehp.9074

**Published:** 2006-07-06

**Authors:** Mercedes Medina-Ramón, Antonella Zanobetti, David Paul Cavanagh, Joel Schwartz

**Affiliations:** 1 Department of Environmental Health and; 2 Department of Epidemiology, Harvard School of Public Health, Boston, Massachusetts, USA

**Keywords:** cause of death, cold, effect modifiers (epidemiology), heat, mortality, temperature, weather

## Abstract

**Background:**

Extremes of temperature are associated with short-term increases in daily mortality.

**Objectives:**

We set out to identify subpopulations and mortality causes with increased susceptibility to temperature extremes.

**Methods:**

We conducted a case-only analysis using daily mortality and hourly weather data from 50 U.S. cities for the period 1989–2000, covering a total of 7,789,655 deaths. We used distributions of daily minimum and maximum temperature in each city to define extremely hot days (≥ 99th percentile) and extremely cold days (≤ 1st percentile), respectively. For each (hypothesized) effect modifier, a city-specific logistic regression model was fitted and an overall estimate calculated in a subsequent meta-analysis.

**Results:**

Older subjects [odds ratio (OR) = 1.020; 95% confidence interval (CI), 1.005–1.034], diabetics (OR = 1.035; 95% CI, 1.010–1.062), blacks (OR = 1.037; 95% CI, 1.016–1.059), and those dying outside a hospital (OR = 1.066; 95% CI, 1.036–1.098) were more susceptible to extreme heat, with some differences observed between those dying from a cardiovascular disease and other decedents. Cardiovascular deaths (OR = 1.053; 95% CI, 1.036–1.070), and especially cardiac arrest deaths (OR =1.137; 95% CI, 1.051–1.230), showed a greater relative increase on extremely cold days, whereas the increase in heat-related mortality was marginally higher for those with coexisting atrial fibrillation (OR = 1.059; 95% CI, 0.996–1.125).

**Conclusions:**

In this study we identified several subpopulations and mortality causes particularly susceptible to temperature extremes. This knowledge may contribute to establishing health programs that would better protect the vulnerable.

Extremes of temperature are associated with short-term increases in daily mortality (Braga et al. 2001; Curriero et al. 2002; Huynen et al. 2001; McGeehin and Mirabelli 2001; Mercer 2003). Identification of factors that confer susceptibility to temperature extremes has recently become an issue of interest in the scientific community. A greater susceptibility has been reported for the elderly and for those with a lower socioeconomic status [Bouchama 2004; Centers for Disease Control and Prevention (CDC) 2004; Curriero et al. 2002; Diaz et al. 2002; O’Neill et al. 2003; Schwartz 2005b]. Little is known, however, regarding medical conditions that may also confer susceptibility, such as diabetes or chronic obstructive pulmonary disease (COPD). A study in Detroit, Michigan, found indications that these conditions, which are increasing in prevalence, may also be relevant factors in determining susceptibility to temperature extremes (Schwartz 2005b). Furthermore, few epidemiologic studies have examined susceptibility considering the cause of death, probably because they had small sample sizes. Recognition of factors that confer susceptibility to mortality from a specific disease on extreme temperature days may provide relevant information for developing public health programs that will better target those most vulnerable.

Here we used a case-only approach to study susceptibility to temperature extremes in 50 U.S. cities. This approach, which examines only decedents, provides important advantages compared with traditional analyses. Advantages of the approach include reduction of potential confounding by variables that are typically associated with mortality (e.g., smoking history, blood pressure), simplification of modeling, and reduction of vulnerability to model mis-specification bias (Armstrong 2003; Schwartz 2005b). Of particular interest is the fact that the seasonal pattern in mortality, whose modeling is quite complex, drops out in this approach (Armstrong 2003; Schwartz 2005b).

The aim of the study was to identify factors that confer susceptibility to extreme temperatures, including sociodemographic characteristics, medical conditions, and place of death, and to explore differences in susceptibility according to the primary cause of death. We also aimed to identify specific mortality causes that experience greater relative increases on extreme temperature days, an issue that has not been addressed by previous studies.

## Materials and Methods

### Study design and population

**Figure 1:**
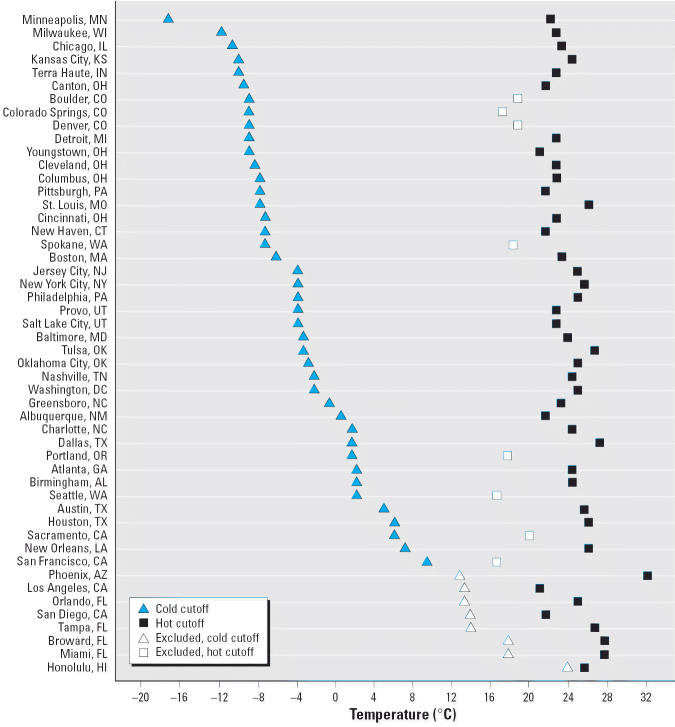
Cutoff points used to define extremely cold days (1st percentile of daily maximum temperature) and extremely hot days (99th percentile of daily minimum temperature) in each of the 50 U.S. cities during the period 1989–2000.

After approval of the study protocol by the Human Subjects Committee from the Harvard School of Public Health, we conducted a case-only analysis using weather and mortality data from 50 U.S. cities during the period 1989–2000. We selected all counties corresponding to the metropolitan areas of cities listed in Figure 1. Cities were randomly chosen from the country’s most populated Metropolitan Statistical Areas (U.S. Census Bureau 1998) with urban counties near appropriate weather stations. A few less-populated cities were also included from census regions that would otherwise have been unrepresented.

The case-only approach was originally proposed to improve efficiency in the study of gene–environment interactions by examining the prevalence of a specific genotype only among cases. As Armstrong suggested (2003), this approach can also be used to investigate how other individual characteristics that do not vary (or vary slowly) over time (e.g., sex or socioeconomic status) modify the effect of a time-varying exposure (e.g., extreme temperatures) on the outcome of interest (e.g., death) by restricting the sample to cases (e.g., decedents). In our study, we applied this approach by comparing the individual characteristics of those dying on extremely hot (or cold) days with those dying on other days. Intuitively, if an individual characteristic increases the risk of dying on extremely hot days, the proportion of decedents presenting that characteristic will be larger on extremely hot days. In other words, extremely hot days should be a predictor of the occurrence of that individual characteristic in death certificates as assessed by logistic regression (Schwartz 2005b). Following this same approach, we also examined whether some causes of death are more susceptible (i.e., increase more) than others to extreme temperatures. Note that in the case-only design, a negative association does not necessarily indicate a decrease in the risk of dying on extreme temperature days for the group of individuals/causes examined. Rather, often this indicates that the increase in the risk of dying associated with extreme temperatures is less pronounced for that group of individuals/causes than for the others.

### Mortality data

We obtained daily mortality data for each city from the National Center for Health Statistics mortality tapes. Individual records included information on primary and secondary causes of death, place of death and personal characteristics such as age, sex, race or educational attainment. Chronic conditions listed in death certificates [codes from the *International Classification of Diseases, 9th revision* (ICD-9) and *10th Revision* (ICD-10)(World Health Organization 1975, 1993)] included diabetes (ICD-9 250; ICD-10 E10-E14) and COPD (ICD-9 490–496, except 493; ICD-10 J40–J44, J47). Examined primary mortality causes included pneumonia (ICD-9 480–487; ICD-10 J12–J18), stroke (ICD-9 430–438; ICD-10 I60–I69), any cardiovascular disease (ICD-9 390–429; ICD-10 I01–I51), myocardial infarction (ICD-9 410; ICD-10 I21, I22) and cardiac arrest (ICD-9 427.5; ICD-10 I46); and secondary causes included congestive heart failure (ICD-9 428; ICD-10 I50) and atrial fibrillation (ICD-9 427.3; ICD-10 I48).

### Environmental data

For each city, we obtained hourly weather data from the nearest National Weather Service surface station (Earthinfo Inc., Boulder, CO) and calculated the daily minimum and maximum temperatures. We defined extremely cold days as those with a daily maximum temperature at or less than the 1st percentile of its distribution in that city. Similarly, we defined extremely hot days as those with a daily minimum temperature at or greater than the 99th percentile. We chose the daily maximum temperature to examine extreme cold and the daily minimum to examine extreme heat because these indicate situations in which there is little relief during the day (for cold) or at night (for heat). The decision to use a city-specific definition for extreme temperatures was based on previous evidence that the relationship between mortality and temperature varies across different regions, with the largest cold effects observed in the warmer regions and vice versa (Braga et al. 2002; Curriero et al. 2002; Eurowinter Group 1997). Because cities in the study presented a wide range of climates, in some cases the cutoff points to define extreme temperatures were in fact set at a quite mild temperature. For that reason, those cities with a cutoff for extreme cold ≥ 10°C or a cutoff for extreme heat ≤ 20°C were excluded from the cold and heat analysis, respectively, leaving a total of 42 cities for each analysis.

We used data from the U.S. Environmental Protection Agency’s Aerometric Retrieval System (Nehls and Akland 1973) to estimate the daily mean concentrations of ozone (8-hr) in 26 cities using an algorithm that averaged levels reported by multiple monitoring locations (Schwartz 2000). Data on particulate matter air pollution were measured irregularly and available only for a small number of cities and therefore were not included in our analyses.

### Statistical analyses

We conducted separate analyses for cold and heat to examine modification of the risk of dying associated to extreme temperatures. In a first stage, we fitted city-specific logistic regression models (PROC LOGISTIC, version 9.1; SAS Institute Inc., Cary, NC) in which the dependent variable was the hypothesized modifier (i.e., either a personal characteristic or a cause of death). The indicator variable for extreme temperature was included as a predictor in the models. Analyses for extremely cold temperatures were conducted only for the cold months (October–April), defined as those months without any extremely hot day in any of the 42 eligible cities. Likewise, the heat analyses were performed only for the warm months (April–October). This was done to exclude hot days from the reference group for extremely cold days and vice versa, thus avoiding an over-or underestimation of the interaction in situations where the modifier of interest acts for both hot and cold days. Although seasonality per se is not a confounder in these models (Schwartz 2005b), if the proposed modifier of the effect of extreme temperature is a modifier of seasonality (e.g., if diabetics have a stronger seasonal pattern in mortality than other persons), confounding with the interaction of interest could occur. Consequently, all models included a sine and cosine term with a 365.24-day period to capture such interactions between season and the characteristic being investigated (Schwartz 2005b). The main effects of season may be quite complex but, as noted by Armstrong (2003), they drop out in a case-only analysis. Thus, we believe a sinusoidal term is sufficient for examining interactions on top of those main effects.

The analyses examining subject characteristics as effect modifiers were repeated separately for deaths due to cardiovascular disease and deaths due to other primary causes. Our baseline analyses focused on extreme temperature during the day of death, because previous studies indicate the largest effect on mortality occurs then (Braga et al. 2001; Curriero et al. 2002). We also repeated all analyses using distributed lag models that included lags 0 and 1 of temperature. Subsequently, for variables significantly modifying the effect of temperature at lag 1, we also fitted distributed lag models including lags 0, 1, and 2. As a sensitivity analysis, we repeated the baseline analyses using different cutoff points to define extreme temperatures. First, we tested the situation when, instead of using the 1st and 99th percentiles of daily maximum and minimum temperatures, we used the respective 5th and 95th percentiles. Then, for the heat analyses, we also tested the use of the 99th percentile of minimum apparent temperature, which is the perceived air temperature given the ambient humidity (Kalkstein and Valimont 1986; Steadman 1979). Finally, because ambient ozone levels tend to peak on hot days (Schwartz 2005a), we repeated the baseline analyses for heat adjusting for ozone exposure on the same day.

In a second stage of analysis, we combined the city-specific results in a random effects meta-analysis assuming that


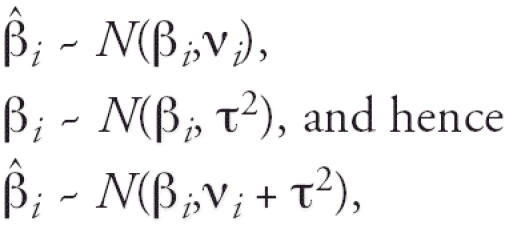


where 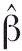 is the effect estimate in city *i*, ν*_i_* is the variance within city *i*, β is the true effect of hot/cold, and τ^2^ is the variance of the true effect between cities, commonly referred to as the random effect. We used the restricted maximum likelihood (REML) procedure of the METAREG function in Stata (version 8; StataCorp, College Station, TX) (Thompson and Sharp 1999) to calculate the overall effect estimate as a weighted average of the city-specific estimates with weights *w_i_* = 1/( ν*_i_* + τ^2^). For the meta-analysis of each effect modifier, cities with < 10 observations in a cell were excluded. We used the *I*^2^ statistic to assess the proportion of total variation in effect estimates that was due to between-cities heterogeneity (Higgins and Thompson 2002). We used the following formula:


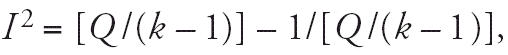


where *Q* is the Q-test for heterogeneity and *k* is the number of cities. Here we present the overall estimates for each effect modifier obtained in the meta-analysis.

## Results

Our analyses included a total of 7,789,655 deaths across 50 U.S. cities. New York City, New York, had the highest number of deaths during the study period, 821,722, whereas smaller cities such as Boulder, Colorado; Provo, Utah; or Terra Haute, Indiana, contributed < 17,000 deaths each. Most of the subjects were > 65 years of age, white, and with a low education (Table 1). A substantial proportion of deaths (42%) occurred out of hospital, and cardiovascular disease (34%) was the most common primary cause of death.

Cities in the study had a wide range of climates, with annual mean temperatures ranging from 7.8°C in Minneapolis, Minnesota, to 25.0°C in Honolulu, Hawaii. Figure 1 shows the cutoff points used in the baseline analysis to define extreme temperatures in each city. Minneapolis had the lowest temperature cutoff for extreme cold (−17.2°C), while Phoenix, Arizona, had the highest temperature cutoff for extreme heat (32.2°C). Figure 1 also shows the cities that were excluded from analyses due to lack of extreme temperatures.

In general, effect modification was modest (Table 2), with the strongest associations observed for extreme heat. Dying outside a hospital was the strongest modifier of the heat effect with an odds ratio (OR) of 1.066 [95% confidence interval (CI). 1.036–1.098]. This effect modification was equally strong and statistically significant for all lags of exposure, with ORs of 1.044 (95% CI, 1.021–1.069), 1.049 (95% CI, 1.026–1.074), and 1.042 (95% CI, 1.015–1.069) for lags 0, 1, and 2, respectively. Other personal characteristics that increased susceptibility to extreme heat in the baseline analysis were old age, diabetes, and black race. All of these were modifiers mainly at lag 0, except for black race, which presented a stronger effect modification at lag 1 (OR = 1.041; 95% CI, 1.02–1.062) and lag 2 (OR = 1.039; 95% CI, 1.020–1.059) than at lag 0 (OR = 1.017; 95% CI, 0.996–1.040). A similar pattern was observed for extreme cold, where black subjects presented a greater susceptibility at lag 1 (OR = 1.021; 95% CI, 1.000–1.043) but not at lag 0 (OR = 1.001; 95% CI, 0.980–1.023). In general, estimates were reasonably homogeneous across cities (*I^2^* < 0.30), except for dying outside a hospital, with 47% and 78% of the total variability in the cold and heat estimates, respectively, being attributable to between-cities differences. Overall, in the sensitivity analysis that used the 5th and 95th percentiles, all associations were similar but with smaller ORs and narrower CIs. For instance, the OR for low-educated subjects was 1.010 (95% CI, 1.001–1.018), compared with 1.016 (95% CI, 0.999–1.033) in the baseline analyses. Results using apparent temperature were also very similar, but did not show evidence for increased heat susceptibility in diabetics (OR = 1.007; 95% CI, 0.979–1.035). Adjustment for ozone level during the warm season resulted in similar, though somewhat lower, estimations (results not shown). The greatest change was observed for the elderly, whose estimate for heat (using the 26 cities with ozone data) changed from an OR of 1.019 (95% CI, 1.003–1.036) to an OR of 1.014 (95% CI, 0.996–1.031) after adjustment.

Table 3 shows the susceptibility of specific causes of death to extreme temperatures. On extremely cold days, the risk of dying from any cardiovascular disease increased more than the risk of dying from other causes (OR = 1.053; 95% CI, 1.036–1.070). This was particularly evident for cardiac arrest (OR = 1.137; 95% CI, 1.051–1.230). Conversely, the increase in the mortality risk associated with extreme heat was smaller for myocardial infarction than for other causes (OR = 0.945; 95% CI, 0.918–0.974). Those having atrial fibrillation showed the highest increase in the risk of dying on extremely hot days; however, results were statistically significant only when the 95th percentile was used instead of the 99th. The rest of the associations were essentially the same when changing the cutoffs to define extreme temperatures, except that cardiac arrest presented a much lower estimate (OR = 1.049; 95% CI, 0.997–1.105) when the 5th percentile was used to define extreme cold, and stroke presented a higher and statistically significant heat estimate when apparent temperature was used (OR = 1.058; 95% CI, 1.026–1.091). In the baseline analysis, heterogeneity of the city-specific estimates was somewhat low, except for the heat estimates for pneumonia and cardiovascular deaths, which presented an *I^2^* of 0.55 and 0.67, respectively. When looking at lags of exposure, all the effect modifications observed in the baseline analysis occurred mainly at lag 0. Adjustment for ozone of the heat baseline models led to essentially the same results with estimates approximately 10% lower (results not shown).

**Figure 2:**
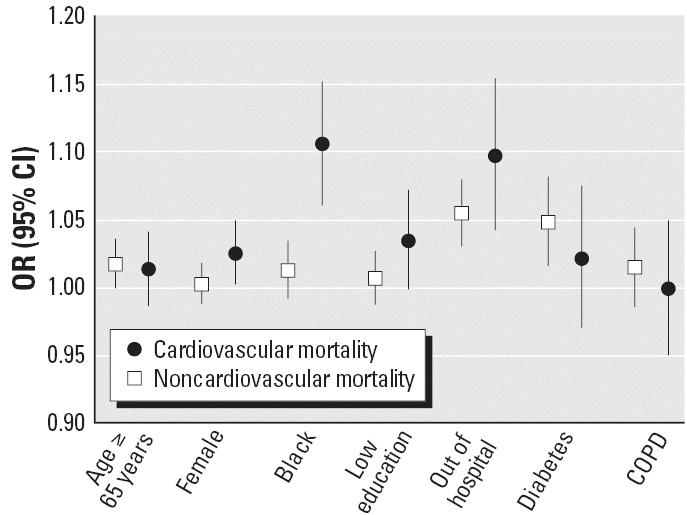
Modification by subject characteristics of the effect of extreme hot temperature on cardiovascular mortality and noncardiovascular mortality: results from the meta-analysis of 42 U.S. cities during the period 1989–2000. Estimates represent the relative odds of dying on an extreme temperature day for persons who had the condition (e.g., being female) compared with persons who did not have the condition.

For some individual characteristics, the effect modification seen in Table 2 differed according to the primary cause of death (Figure 2). The increased susceptibility of diabetics to extreme heat, for instance, was larger and statistically significant when the primary cause of death was noncardiovascular (OR = 1.048; 95% CI, 1.014–1.082), whereas black subjects were more susceptible when their cause of death was cardiovascular (OR = 1.105; 95% CI, 1.059–1.152). In this last instance, however, differences according to the primary cause of death became less evident in the distributed lag models and also when using the 95th percentile cutoff in the sensitivity analysis. Those dying outside a hospital were more susceptible to extreme heat regardless of the primary cause of death, whereas their susceptibility to extreme cold was only increased when the primary cause of death was cardiovascular (OR = 1.062; 95% CI, 1.025–1.101; results not shown). Finally, females and low-educated subjects dying from a cardiovascular disease showed a marginally increased susceptibility to extreme heat, which became more pronounced at lag 1 in the distributed lag model for both females [ORs = 1.013 (95% CI, 0.989–1.039) and 1.033 (95% CI, 1.005–1.061) for lags 0 and 1, respectively] and low-educated subjects [ORs = 1.018 (95% CI, 0.986–1.052) and 1.043 (95% CI, 1.010–1.078) for lags 0 and 1, respectively].

## Discussion

In a large, multicity study we identified sub-populations with increased sensitivity to extreme temperatures. Black subjects, those ≥ 65 years of age, and diabetics were especially vulnerable to extreme heat, with some differences in susceptibility observed between those dying from a cardiovascular disease and those dying from other causes. The increase in deaths on extreme temperature days was significantly higher for out-of-hospital deaths than for inhospital deaths. Regarding specific mortality causes, cardiovascular deaths showed a higher susceptibility to extreme cold, which was particularly noticeable for cardiac arrest deaths. Another interesting finding was the marginally increased vulnerability conveyed by atrial fibrillation, which has not been previously reported.

Our results suggest that advanced age increases susceptibility to temperature extremes, with a more marked effect for heat. Several studies on the effects of temperature on mortality have also found a greater susceptibility of the elderly to both cold (Curriero et al. 2002; O’Neill et al. 2003) and hot temperatures (Bouchama 2004; CDC 2004; Curriero et al. 2002; Fish et al. 1985; Greenberg et al. 1983; Grize et al. 2005; Johnson et al. 2005; Jones et al. 1982; Ramlow and Kuller 1990; Whitman et al. 1997). A reduced thermoregulatory capacity in the elderly, combined with a diminished ability to detect changes in their body temperature, may partly explain their increased susceptibility (Mercer 2003), although other physiologic differences in nonthermoregulatory responses to extreme temperatures may also play a role (Smolander 2002).

Our results showed that blacks and low-educated subjects, which may be predictors of low socioeconomic status, were more susceptible to extreme heat. A greater susceptibility to die on hot days has been previously reported for blacks (Applegate et al. 1981; Greenberg et al. 1983; O’Neill et al. 2003; Schwartz 2005b; Whitman et al. 1997), the low-educated (O’Neill et al. 2003), and those with low socioeconomic status (Applegate et al. 1981; CDC 2004; Greenberg et al. 1983) and could be related to poorer health status, limited access to health care, and poorer housing conditions in these socially disadvantaged groups (Marmot and Feeney 1997; McGeehin and Mirabelli 2001). We also found that those dying outside a hospital were more susceptible to extreme temperatures, especially to heat. This finding agrees with those of previous studies (CDC 2004; O’Neill et al. 2003) and supports the hypothesis that ambient extreme temperatures affect rates of mortality.

When examining whether some underlying chronic conditions increase the susceptibility to extreme temperatures, we confirmed the finding of Schwartz (2005b) that diabetics are particularly vulnerable to heat. This could be explained by a different response to extreme thermal stress in diabetics, which may be determined by their impairment of the autonomic control and endothelial function. On the other hand, although one might expect that individuals with COPD would show an increased susceptibility to cold temperatures, we were unable to replicate that finding from Schwartz (2005b), perhaps because we identified COPD from death certificates, whereas he identified subjects with COPD noted on hospital discharge records.

An important finding of our study is that susceptibility to temperature extremes varies according to the primary cause of death. Few studies have looked at susceptibility to temperature stratifying by cause of death. A study in seven U.S. cities found that younger subjects, blacks, and those dying outside a hospital were more susceptible to extreme cold when they died from a respiratory disease, but observed no differences in susceptibility for those dying from a cardiovascular disease (O’Neill et al. 2003). In contrast, we found evidence that the increased susceptibility of black subjects to heat was more pronounced when they died from a cardiovascular disease, as it was for females and low-educated subjects. On the other hand, older subjects and diabetics were more susceptible to heat when their primary cause of death was not cardiovascular. These differences in susceptibility according to the cause of death are relevant to more specifically target populations and identify health services and infrastructures necessary to reduce the impact of extreme temperatures on mortality.

Some evidence in the literature shows that cardiovascular deaths increase during both extremely cold days and extremely hot days (Applegate et al. 1981; Braga et al. 2002; Curriero et al. 2002; Kalkstein and Greene 1997; Kunst et al. 1993; Mercer 2003). In our study, we found evidence that the increase in cardiovascular deaths during extremely cold days was significantly higher than that of other mortality causes. Mechanisms that may explain such a marked increase in cardiovascular deaths with exposure to extreme cold have been postulated. Blood pressure is higher during the winter (Imai et al. 1996; Woodhouse et al. 1993) and it has been reported that exposure to cold temperatures increases levels of plasma cholesterol and plasma fibrinogen (Keatinge et al. 1984; Neild et al. 1994; Stout and Crawford 1991), which could lead to thrombosis through haemoconcentration. On the other hand, we found indications that those with coexisting atrial fibrillation may be particularly vulnerable to the effects of extreme heat. Atrial fibrillation may contribute to death in several ways, including increasing the risk of stroke up to 5-fold (Wolf et al. 1991). An increase in blood viscosity and cholesterol levels with high temperatures (Keatinge et al. 1986) may interact with atrial fibrillation to facilitate the formation of blood clots that cause stroke. This mechanism is also consistent with the marginal relative increase observed in stroke deaths during extremely hot days. However, it is important to remember that only about 15–20% of strokes are related to atrial fibrillation (Go et al. 2001).

Given that we used a city-specific definition to determine exposure to extreme temperatures, the fact that effect modification was fairly homogeneous across cities for most of our proposed modifiers, particularly for cold effects, supports our use of a relative (rather than absolute) temperature and agrees with previous studies suggesting that populations tend to adapt to the local climate (Braga et al. 2002; Curriero et al. 2002; Eurowinter Group 1997; Keatinge et al. 2000). This adaptation may occur either by physiologic acclimatization, behavioral patterns, or other adaptive mechanisms, such as having heating or air conditioning at home (Eurowinter Group 1997; Keatinge et al. 2000). The effects of a possible increase in global temperature due to climate change may be partly mitigated by some of these adaptive mechanisms, but extreme temperatures (i.e., unusually high or low for the local climate) will still occur. Thus, recognition of subpopulations that are particularly vulnerable to temperature extremes is of public health relevance, especially if such subpopulations—as is the case of diabetics and the elderly in many countries (CDC 2004; Schwartz 2005b)—are growing proportions of the population.

One limitation of our study is that, due to its case-only design, the excess risk of dying associated with extreme temperatures in each subpopulation or mortality-cause category was not estimated in absolute terms, but relative to the excess risk in other subpopulations/causes. Consequently, it was not possible to ascertain whether the negative association for myocardial infarction represented a decrease in risk on hot days or a less pronounced increase compared with other mortality causes. The most likely scenario is the latter, given previous epidemiologic evidence of an increase in the risk of myocardial infarction on hot days (Braga et al. 2002). Also, although we adjusted our results for ambient ozone levels, we did not have adequate data available to consider exposure to particulate matter, which constitutes another limitation of the study. If exposure to particulate matter peaked on extreme temperature days and some of the subpopulations studied were particularly vulnerable to the effects of particulate matter, then our results may have been overestimated. Also, using data from a mortality registry is likely to result in some misclassification of death cause and particularly some underreporting of contributing causes of death. This underreporting and misclassification will at a minimum reduce statistical power and induce some downward bias in the regression coefficients. Although, *a priori*, this misclassification should be unrelated to ambient temperature, this may not be the case for diagnoses, such as cardiovascular disease, that have been consistently related to cold temperatures in the past. Finally, because we focused on extreme temperature days, our results cannot be extrapolated to moderately hot or cold temperatures.

In conclusion, we confirmed in a large sample of cities that subpopulations such as the elderly, diabetics, and black subjects are especially susceptible to temperature extremes. According to our results, susceptibility of populations may vary according to the primary cause of death, suggesting that future studies on susceptibility will benefit from taking this into consideration. Finally, we found evidence that cardiovascular deaths, especially cardiac arrest deaths, show much larger increases on extremely cold days than other mortality causes.

## Figures and Tables

**Table 1:** Baseline descriptive statistics for deaths (*n* = 7,789,655) in 50 U.S. cities during the period 1989–2000.

	Percent of total deaths	
Characteristic	Any occurrence	Primary cause of death
Age ≥ 65 years	68.2	—
Female	48.7	—
Race		
Black	18.9	—
White	78.2	—
Low education^a^	74.4	—
Out-of-hospital death^b^	41.6	—
Presenting condition		
Diabetes	7.7	2.4
COPD	8.1	3.7
Pneumonia	9.1	3.5
Stroke	10.9	6.2
Cardiovascular disease	55.0	34.1
Myocardial infarction	9.8	8.4
Cardiac arrest	19.8	0.8
Congestive heart failure	10.3	1.5
Atrial fibrillation	1.9	0.2

a High school graduate or less; percentage based on 6,647,937 observations.

b Percentage based on 7,638,789 observations.

**Table 2:** Modification by subject characteristics of the effect of extreme temperatures on mortality.^a^

	Extreme cold	Extreme heat
Characteristic	OR (95% CI)	OR (95% CI)
Sociodemographic characteristics		
Age ≥ 65 years	1.018 (0.998–1.039)	1.020 (1.005–1.034)
Female	0.998 (0.983–1.013)	1.011 (0.997–1.024)
Black race	1.009 (0.990–1.029)	1.037 (1.016–1.059)
Low education^b^	1.006 (0.983–1.030)	1.016 (0.999–1.033)
Out-of-hospital death	1.020 (0.995–1.046)	1.066 (1.036–1.098)
Presenting chronic condition		
Diabetes	0.979 (0.951–1.008)	1.035 (1.010–1.062)
COPD	0.995 (0.968–1.023)	1.004 (0.979–1.030)

a Results from the meta-analysis of 50 U.S. cities during the period 1989–2000. Estimates represent the relative odds of dying on an extreme temperature day for persons who had the condition (e.g., being female) compared with persons who did not have the condition.

b High school graduate or less.

**Table 3:** Primary and contributing causes of death as modifiers of the effect of extreme temperatures on mortality.^a^

	Extreme cold	Extreme heat
Cause	OR (95% CI)	OR (95% CI)
Primary cause of death		
Pneumonia	1.028 (0.979–1.079)	1.008 (0.944–1.076)
Stroke	0.987 (0.956–1.020)	1.026 (0.997–1.055)
Cardiovascular disease	1.053 (1.036–1.070)	1.010 (0.985–1.037)
Myocardial infarction	1.030 (0.999–1.062)	0.945 (0.918–0.974)
Cardiac arrest	1.137 (1.051–1.230)	0.971 (0.897–1.051)
Contributing cause of death		
Congestive heart failure	0.976 (0.947–1.005)	0.981 (0.954–1.009)
Atrial fibrillation	1.052 (0.993–1.115)	1.059 (0.996–1.125)

a Results from the meta-analysis of 50 U.S. cities during the period 1989–2000. Estimates represent the relative odds of dying on an extreme temperature day due to the examined condition (e.g., having a myocardial infarction) compared with dying from other causes.
